# Validation and refinement of the Stakeholder-driven Community Diffusion Survey for childhood obesity prevention

**DOI:** 10.1186/s13012-021-01158-4

**Published:** 2021-10-09

**Authors:** Ariella R. Korn, Julia Appel, Ross A. Hammond, Erin Hennessy, Louise C. Mâsse, Aviva Must, Christina D. Economos

**Affiliations:** 1 Friedman School of Nutrition Science and Policy, Tufts University, Boston, MA USA; 2 Cancer Prevention Fellowship Program, Implementation Science, Office of the Director, Division of Cancer Control and Population Sciences, National Cancer Institute, 9609 Medical Center Drive, Rockville, MD 20850 USA; 3 Center on Social Dynamics and Policy, Brookings Institution, Washington, DC USA; 4 Brown School at Washington University, St. Louis, MO USA; 5 BC Children’s Hospital Research Institute, School of Population and Public Health, University of British Columbia, Vancouver, BC Canada; 6 Public Health and Community Medicine, Tufts University School of Medicine, Boston, MA USA

**Keywords:** Childhood obesity prevention, Community settings, Confirmatory factor analysis, Psychometric evaluation, Survey development, Whole-of-community interventions

## Abstract

**Background:**

Whole-of-community interventions hold promise in addressing childhood obesity. The COMPACT Stakeholder-driven Community Diffusion theory posits that stakeholders’ knowledge of childhood obesity prevention efforts and engagement with the issue contribute to successful intervention implementation. Building on completed formative research and pilot testing, we describe the validation and refinement of knowledge and engagement measures.

**Methods:**

We assessed content validity using a modified Delphi process with science (*n*=18) and practice-based (*n*=16) experts. Next, we refined the survey based on input from science- and practice-based experts, cognitive response testing, and item analysis of extant survey data. Field testing of the refined survey involved community stakeholders in Greenville County, South Carolina (*n*=50), East Boston, Massachusetts (*n*=30), and Tucson, Arizona (*n*=84) between 2019 and 2020. Construct validity was assessed with confirmatory factor analysis (CFA). Two-week test-retest reliability was assessed among a subsample of 14 paired respondents in South Carolina.

**Results:**

Experts rated existing knowledge domains (*intervention factors*, *roles*, *sustainability*, *problem*, *resources*) and engagement domains (*dialogue/mutual learning*, *flexibility*, *influence/power*, *leadership/stewardship*, *trust*) highly for their importance in addressing childhood obesity. Expert input resulted in 11 new knowledge items and 7 new engagement items that mapped onto existing domains. Correspondingly, two domain names were modified: *implementation/sustainability* and *trust/trustworthiness*. We also eliminated 8 extant items (4 knowledge and 4 engagement) and adapted item language for comprehension and consistency. Further modifications based on CFA results and item analyses resulted in 23 knowledge items across four domains (*roles* and *resources* merged) and 23 engagement items across five domains. Modified knowledge and engagement scales had adequate fit and strong item factor loadings (most >0.7 and all >0.5). Knowledge (*α*=0.86–0.87) and engagement (*α*=0.75–0.90) subscales had high internal scale consistency. Knowledge intraclass correlation coefficients (ICCs) for test-retest agreement of subscale scores ranged from 0.50 for *intervention factors* to 0.86 for *roles/resources*. For engagement subscale scores, ICCs ranged from 0.70 for *trust/trustworthiness* to 0.96 for *leadership/stewardship*.

**Conclusions:**

Findings from this multi-method survey development process increase our confidence of the knowledge and engagement measures’ content validity, construct validity, and reliability.

**Supplementary Information:**

The online version contains supplementary material available at 10.1186/s13012-021-01158-4.

Contributions to the literature

- Applied researchers need valid, reliable, pragmatic, and context-specific measures to understand the dynamics of implementing evidence-based interventions that address childhood obesity in community settings.
- Building on prior work, we validated and refined two scales: community stakeholders’ “knowledge” of childhood obesity prevention efforts and their “engagement” with the issue. Both are multi-dimensional constructs grounded in theory and previous evidence.
- Our study demonstrates the process and the value of input from science- and practice-based experts, iterative refinement, and field testing among stakeholders in three U.S. communities.
- The modified knowledge and engagement scales (23 items each) have strong reliability and validity characteristics.

## Background

Decades of childhood obesity research offer a strong understanding of potential practice, policy, and environmental changes at the community-level that can promote children’s obesity-preventive behaviors and obesity-related outcomes [1–3]. However, interventions are routinely challenged by low adoption, coordination, and lack of sustainability, significantly decreasing their potential to improve children’s health [4]. Enhancing the success of childhood obesity prevention interventions may require an “upstream” shift by focusing on the stakeholders (e.g., caregivers, healthcare providers, and local government officials) who influence implementation efforts and shape children’s behavioral and biologic development.

To address this gap, the Childhood Obesity Modeling for Prevention and Community Transformation (COMPACT) study developed and is testing a novel theory of “Stakeholder-driven Community Diffusion” in the context of whole-of-community childhood obesity prevention interventions (R01HL115485) [5–9]. In this theory, stakeholders’ *knowledge* of childhood obesity and how to address it and their *engagement* with the issue diffuse throughout their *social networks*. We expect that this diffusion process supports the planning, successful implementation, and sustainability of whole-of-community childhood obesity prevention efforts.

Reliable, valid, and context-specific measures are required to operationalize and test the Stakeholder-driven Community Diffusion theory. Our prior work describes the development and pilot testing of initial knowledge, engagement, and social network measures [5]. Using naming conventions based on software release lifecycles, the “alpha prototype” (v1) was tested retrospectively in 2015 with stakeholders involved in two completed whole-of-community interventions: Shape Up Somerville (Somerville, Massachusetts, U.S.) [10] and Romp & Chomp (Geelong, Victoria, Australia) [7, 11]. Following refinement for prospective use, the “beta prototype” (v2) included 18 knowledge items across five domains (*intervention factors*, *roles*, *sustainability*, *problem*, *resources*), 25 engagement items across five domains (*dialogue & mutual learning*, *flexibility*, *influence & power*, *leadership & stewardship*, *trust*), and 5 items to assess stakeholders’ network structure (using name generator and name interpreter questions commonly applied in survey-based social network research [12]). The beta prototype knowledge and engagement scales demonstrated good test-retest reliability and internal scale consistency among 13 Australian respondents [5]; however, the beta prototype scales had not yet been tested in the U.S. among larger samples nor evaluated for further psychometric properties to inform their validity. Therefore, further validation work was required to achieve our goal of developing and disseminating psychometrically sound measures to potentially improve the planning, implementation, and sustainability of whole-of-community efforts to prevent childhood obesity.

This paper describes a multi-method survey validation and refinement process focused on the COMPACT Stakeholder-driven Community Diffusion Survey’s knowledge and engagement measures.

## Methods

**Fig. 1:**
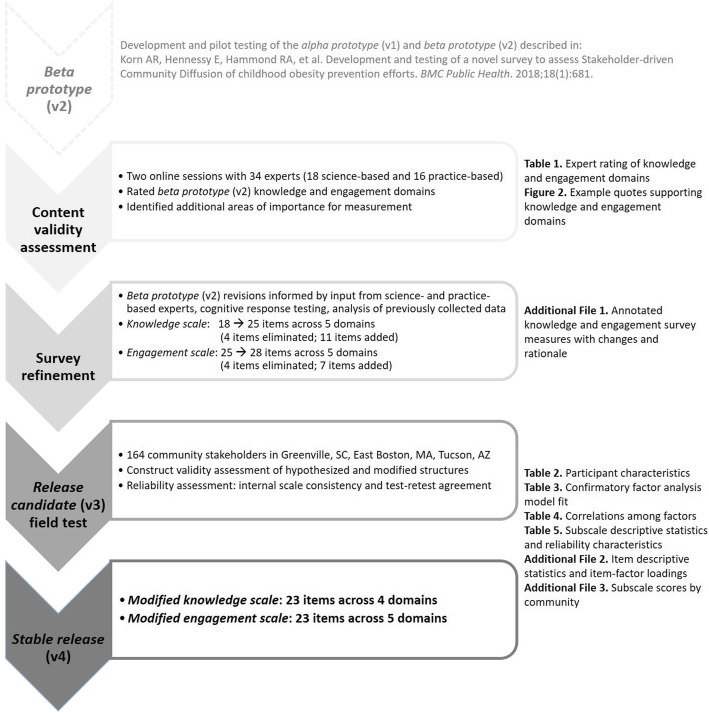
Overview of the validation and refinement of the COMPACT Stakeholder-driven Community Diffusion Survey

We completed this study in three phases (Fig. 1). First, we assessed the content validity of the knowledge and engagement measures with science- and practice-based experts. Second, we refined the survey based on expert input, cognitive response testing, and item analysis of previously collected survey data with the beta prototype (v2). Third, we field tested the refined “release candidate” (v3) survey to inform the knowledge and engagement measures’ construct validity and reliability. All study procedures were approved by the Tufts University Institutional Review Board.

### Content validity assessment

We sought input from science- and practice-based experts to substantiate the content of existing knowledge and engagement measures and to inform revisions to these measures.

#### Participants

We first identified science-based experts in disciplines such as childhood obesity prevention, community psychology, organizational theory, implementation science, and community-based participatory research—fields chosen to elicit comprehensive content, theoretical, and applied expertise. Sampling was informed by literature reviews, identification of individuals who were lead/senior authors of relevant articles and systematic reviews, authorship indices, and the research team’s professional networks. We also identified practice-based experts (i.e., multisector community leaders, professionals, practitioners working to prevent childhood obesity and promote child health) through nominations made by participating science-based experts and the research team. This process helped to achieve greater geographical diversity in the study sample of practice-based experts. Prior Stakeholder-driven Community Diffusion Survey respondents were ineligible to participate.

#### Recruitment

For both expert groups, we recruited participants by email and/or telephone starting approximately 1.5 months in advance of the pre-determined session dates to avoid scheduling conflicts. Upon recruitment, participants in both groups were provided a description of the Stakeholder-driven Community Diffusion theory and referred to our prior survey development publication [5] for additional information. Participants were offered a gift card for their participation and were sent a summary report following completion of data collection.

#### Data collection procedures

We commissioned the market research firm Advanced Strategy Center (Scottsdale, Arizona, U.S.) to engage science- (Fall 2018) and practice-based (Spring 2019) experts in two 90-min participatory online sessions (one per expert group) on the firm’s proprietary platform. Both sessions followed the same data collection procedures. The sessions were professionally facilitated by Advanced Strategy Center using a detailed guide designed by the research team. Participants joined the session via web browser (using a unique log-in ID) and also by telephone to listen to the verbal facilitation. All responses were provided electronically and anonymously. Members of the research team joined both sessions to verbally welcome participants and support the facilitator—for example, by helping to identify themes from open-ended responses (described below).

Following a few sociodemographic questions, the sessions began by asking participants a series of open-ended framing questions about the issue of childhood obesity (“Why is this issue so important to you? Why does it matter—what’s at stake?”), strategies to address it (“What strategies and approaches come to mind across various setting and sectors?”), and identification of key stakeholders (“Please list the types of stakeholders that come to mind when thinking about community-level childhood obesity prevention.”).

Participants then completed a series of activities—first about knowledge concepts and then about engagement concepts—as follows:

i. Participants reviewed existing knowledge domains (*intervention factors*, *roles*, *sustainability*, *problem*, *resources*) and engagement domains (*dialogue & mutual learning*, *flexibility*, *influence & power*, *leadership & stewardship*, *trust*) and their definitions (see Table 1).
ii. Participants rated each domain “in terms of how important you feel it is in catalyzing community change related to childhood obesity prevention” on a scale of 1 (“not at all important”) to 10 (“extremely important”).
iii. Participants identified additional areas of importance related to knowledge and engagement concepts that were not already captured, with probes such as “What kinds of knowledge, content expertise, or understanding do stakeholders need to address childhood obesity?” and “Are there other characteristics related to group dynamics that are important for stakeholders in addressing childhood obesity?”
iv. The open-ended responses generated in (iii) were consolidated into themes. Participants then rated each theme based on their perceived importance using the same prompt as (ii).

Next, participants were asked to identify “other characteristics or stakeholder assets that are important in addressing childhood obesity” beyond knowledge and engagement concepts previously discussed. These open-ended responses were consolidated into themes, and then participants selected one theme of greatest importance and provided their rationale. The sessions concluded with opportunities to provide feedback, recommendations, and questions for the research team moving forward.

For most questions, the proprietary platform allowed participants to compare their responses to that of the group by displaying individual responses on the screen anonymously in real time (apart from sociodemographic questions that could compromise anonymity). We used this approach, rather than a traditional Delphi method [13], to reduce participant burden and avoid attrition across multiple rounds of surveys.

#### Data analysis

For each session and combined, we calculated frequencies for categorical items and median (range) for ordinal data. We also conducted content analysis with all open-ended responses.

### Survey refinement

We refined the beta prototype (v2) survey with information from three sources: (i) survey cognitive response testing, (ii) previously collected beta prototype knowledge and engagement survey data, and (iii) findings from the content validity assessment. Through this refinement process, we also had an eye toward decreasing participant burden by abbreviating the knowledge and engagement scales.

#### Survey cognitive response testing

In summer 2018, we conducted cognitive interviews to understand respondents’ interpretation of items, response processes, and sources of potential response error [14] for beta prototype (v2) knowledge and engagement survey modules. Potential participants included a convenience sample of prior survey respondents from the Shape Up Under 5 pilot whole-of-community intervention (Somerville, Massachusetts; 2015–2017) [6, 8, 9]. Recruitment strived for diverse representation of organizational sectors and professional backgrounds related to early childhood health. Participants were recruited via email and/or telephone using contact information acquired during the Shape Up Under 5 study and were offered a gift card for their participation in the interview.

Interviews were completed in-person and audio-recorded. To simulate the intended web-based self-administered survey procedure in which respondents answer questions unaided, we utilized a hybrid design. Participants completed the survey on a laptop page-by-page, with approximately five items per page. After each page was completed, participants were verbally probed about those items to assess item and terminology comprehension, the ability to recall information confidently, and items’ level of difficulty [14]. For example, for the knowledge item *I know strategies to prevent obesity in early childhood that can be sustained over time*, potential probes included “How did you arrive at your answer?”, “What does the term ‘sustained’ mean to you?”, “What time period are you thinking of for ‘over time’”?, and “Would it be easier or harder to answer this statement if it included a specific time period, for example, 10 years?” Analysis involved detailed review of interviewer notes and audio recordings for each knowledge and engagement item, annotation of proposed changes and rationale, and discussion with members of the research team.

#### Examining previously collected data

Survey refinement was also informed by knowledge and engagement data collected prospectively with the beta prototype (v2) survey [5] in Somerville, Massachusetts [6] and Cuyahoga County, Ohio, between 2015 and 2019. Manuscripts describing knowledge and engagement results in these communities are forthcoming. We examined mean±SD responses of the 18 knowledge items and 25 engagement items from a total of 300 observations in Somerville and 239 observations in Cuyahoga County. Observations were stratified by community and measurement period. We reviewed items using the following criteria derived from extant measurement research [15–19]:

- *Limited response variability* in > 1 measurement period in both community sites, defined conservatively as a SD < 10% of the maximum response value.
- *Limited change* in mean scores (< 5%) across measurement rounds.
- *Low item-total correlations* (< 0.4) in both community sites.

Items that met any of the above criteria were considered for elimination from the survey. However, if an item captured concepts salient in the content validity assessment, then it was retained.

#### Integrating input from science- and practice-based experts

Using findings from the content validity assessment, we considered the addition of new measurement constructs (distinct from knowledge and engagement), new domains within knowledge and engagement, and new survey items that fit within existing constructs (when possible, adapted from existing measures) for the refined release candidate (v3) survey. We prioritized concepts salient to both science- and practice-based experts. We also considered concepts salient to one expert group if it was reported by multiple participants and/or rated highly. All considered additions were reviewed by the research team and corroborated with the literature on childhood obesity prevention, community engagement, and related fields.

### Field test and assessment of measurement properties

We administered the refined release candidate (v3) survey with stakeholders in three U.S. communities to inform the measures’ reliability and construct validity.

#### Participants and procedures

The refined survey was administered via Qualtrics among stakeholders involved in childhood obesity prevention efforts in partnership with the research team in Greenville County, South Carolina (Fall 2019), East Boston, Massachusetts (Fall 2019), and Tucson, Arizona (Fall 2020) as part of the “Catalyzing Communities” initiative [20]. Each project was led by a core group of multisector stakeholders (e.g., healthcare, education, local government) that ranged in size from 11 (Tucson) to 19 (Greenville County) members. In each community, we used a snowball sampling approach that leveraged connections of core group members to field test the survey among a broader group of multisector stakeholders. Survey distribution in Tucson additionally leveraged listservs and coalitions focused on child and community health and wellbeing.

Following a convenience sampling approach, core group members in Greenville County were invited to complete a second survey to inform the knowledge and engagement measures’ two-week test-retest reliability. Surveys were administered at each project’s respective baseline.

#### Data analysis

Analyses were conducted in RStudio (version 1.2.5019) and Mplus (version 8.4) among complete knowledge and engagement responses.

#### Construct validity and scale modifications

We conducted confirmatory factor analyses (CFA) to test hypothesized knowledge and engagement measurement structures, each with five domains (latent factors) and items (indicator variables) that load on each factor. We evaluated model fit with multiple indicators: chi-square test and degrees of freedom (df), root mean square error of approximation (RMSEA) and its corresponding 90% CI (<0.05 good fit and upper CI <0.08), standardized root mean square residual (SRMR) (<0.05 good fit, <0.08 adequate fit), Comparative Fit Index (CFI) (>0.95 good fit, >0.90 adequate fit), and Tucker-Lewis Index (TLI) (>0.95 good fit, >0.90 adequate fit) [21]. We made incremental structural changes to the knowledge and engagement scales—for example, by removing an item or combining factors—using multiple information sources, including results from the CFA analysis (striving for aforementioned model fit indicators), conceptual evaluation of items, and response variability. Our goal was to improve model fit without compromising theory and conceptual rationale. In keeping with the software naming convention, we refer to the modified scales as “stable release” (v4).

#### Scores

Using the modified knowledge and engagement scales, we calculated mean±SD and median (range) responses of each item. Domain (i.e., subscale) scores were calculated for each observation by averaging responses of items within their respective domain (maximum of 5 points). We then calculated mean±SD subscale scores across observations. We used Kruskal-Wallis rank sum tests to explore differences in scores across communities.

#### Reliability

Using the modified knowledge and engagement scales, we assessed the internal consistency of knowledge and engagement subscales using Cronbach’s alpha statistic. Further, we assessed absolute agreement in subscales scores across test-retest surveys with intraclass correlation coefficients (ICCs). ICC estimates were based on multiple observers and two-way mixed-effects models [22] and are commonly interpreted as < 0.40 “poor”, 0.40–0.59 “fair”, 0.60–0.74 “good”, and 0.75–1.00 “excellent” [23]. We also calculated within-subject coefficient of variation (WSCV) and corresponding 95% confidence intervals (CIs) as an alternative measure of test-retest reliability. WSCV estimates are interpreted as the average percentage of variation between test and retest scores among paired respondents.

## Results

### Content validity assessment

#### Participant characteristics

We invited 101 science-based experts to participate, of which 25 declined, 56 did not respond, and 18 participated (17.8%). Participants represented 16 U.S. institutions across 11 states. Participants were mostly female (*n*=16; 88.9%), on average 50 years old (range 33–66 years), and reported primary or secondary expertise in obesity prevention (*n*=13, 72.2%), community-based participatory research (*n*=9, 50.0%), health policy (*n*=4, 22.2%), community psychology (*n*=3, 16.7%), health and medicine (*n*=2, 11.1%), implementation science (*n*=2, 11.1%), nutrition (*n*=1, 5.6%), social networks (*n*=1, 5.6%), and other (*n*=1, 5.6%). Most participants (*n*=15, 83.3%) reported having more than 10 years of professional experience these areas of expertise.

Sixteen practice-based experts participated in the second session (total invitations unknown due to human subjects research protection requirements of the nomination-based sampling). Participants were from the U.S. (*n*=15, 93.8%) and Canada (*n*=1, 6.3%), mostly female (*n*=11, 68.8%), on average 49 years old (range 24–81 years), and represented community-based organizations (*n*=7, 43.8%), local government (*n*=3, 18.8%), community-based research organizations (*n*=3, 18.8%), childcare (*n*=2, 12.5%), and healthcare (*n*=1, 6.3%). Most participants (*n*=9, 56.3%) reported having 5–10 years of experience in their fields.

#### Rating existing measurement domains

The pre-defined knowledge and engagement domains were rated highly by both expert groups (Table 1). For knowledge, median ratings among all participants were 8 out of 10 points for the *intervention factors*, *sustainability*, *problem*, and *resources* domains and 9 points for the *roles* domain. For engagement, median ratings for all domains (*dialogue & mutual learning*, *flexibility*, *influence & power*, *leadership & stewardship*, *trust*) were 9 points on the 10-point scale.

**Table 1:** Expert rating of knowledge and engagement domains from content validity assessments

Domain	Science-based experts (***n***=18)		Practice-based experts (***n***=15)^a^		Combined (***n***=33)	
	Median	Range	Median	Range	Median	Range
**Knowledge**						
***Intervention factors*:** Stakeholders’ knowledge of modifiable determinants of childhood obesity and level of social ecology to address them (e.g., individual-level versus policy-level)	8	6–10	8	7–10	8	6–10
***Roles*:** Stakeholders’ knowledge of their role in the intervention, what others are doing, and multi-setting components (e.g., healthcare, childcare)	9	7–10	9	6–10	9	6–10
***Sustainability*:** Stakeholders’ knowledge of how to intervene to achieve sustainability over time	7.5	2–10	8^b^	6–10^b^	8	2–10
***Problem*:** Stakeholders’ knowledge of the problem of childhood obesity	6.5	2–10	8	3–10	8	2–10
***Resources*:** Stakeholders’ knowledge of available resources to address childhood obesity	8	3–10	9	6–10	8	3–10
**Engagement**						
***Dialogue & mutual learning*:** Stakeholders’ exchange of skills and understanding	9	5–10	10	3–10	9	3–10
***Flexibility*:** Stakeholders’ willingness to compromise and adapt	8	7–10	9	3–10	9	3–10
***Influence & power*:** Stakeholders’ ability or capacity to have an effect on a course of events, others’ thinking, and behavior	9	5–10	9	6–10	9	5–10
***Leadership & stewardship*:** Stakeholders’ action of directing and being responsible for a group of people or course of events	9.5	6–10	9	7–10	9	6–10
***Trust*:** Stakeholders’ belief and confidence in others	9.5	7–10	9	3–10	9	3–10

Each domain was rated on a scale from 1 (not at all important) to 10 (extremely important) in catalyzing community change related to childhood obesity prevention. Two domain names were modified after incorporating expert input (as presented in Tables 4 and 5): *sustainability* to *implementation & sustainability* and *trust* to *trust & trustworthiness*

^a^While *n*=16 practice-based experts participated in the session overall, *n*=15 responded to these set of questions

^b^*n*=14 practice-based experts respondents

**Fig. 2:**
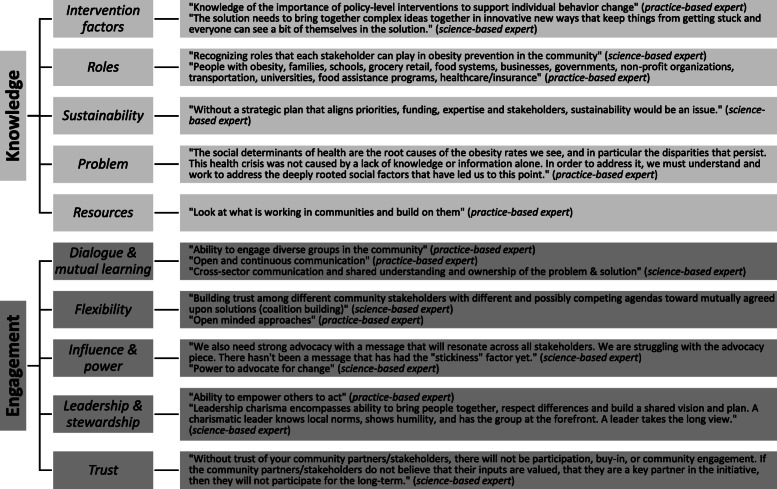
Example quotes supporting knowledge and engagement domain concepts from content validity assessments. Two domain names were modified after incorporating expert input (as presented in Tables 4 and 5): *sustainability* to *implementation & sustainability* and *trust* to *trust & trustworthiness*

Figure 2 includes example quotes from participating science- and practice-based experts that support knowledge and engagement domain concepts. Below we describe how these experts’ input informed specific survey revisions.

### Survey refinement

Additional File 1 includes annotated knowledge and engagement measures with documented changes to the beta prototype (v2) survey during the refinement process, resulting in the release candidate (v3) used in the field test (described below).

#### Survey cognitive response testing

We completed nine interviews (64% response rate). Participants represented a variety of settings and sectors including healthcare, early education and care, local government, and community-based organizations. Most participants were female (*n*=7), non-Hispanic White (*n*=7), and had a Master’s degree or higher level of education (*n*=6). On average, participants reported having 22 years of experience in their respective fields (range 10–43 years).

Specific language changes to improve item comprehension and reduce response error are documented in Additional File 1. Broadly, findings indicated a need for the following modifications: definitions throughout the survey and instructions (e.g., childhood obesity prevention “strategies” and the ages included in “early childhood”); consistent item formats (e.g., “I statements”); consistent item anchoring to the issue of childhood obesity prevention, rather than general statements of characteristics like leadership and flexibility, as we expected that measures will be more sensitive to change over time and more actionable as possible intervention targets; and more explicit instructions that respondents should answer survey items in a professional context, rather than as a parent of a toddler, for example.

#### Examining previously collected data

We identified 14 items for exclusion from the beta prototype (v2) knowledge (5 items) and engagement (9 items) measures that were administered in Somerville and Cuyahoga County (Additional File 1). We eliminated 4 knowledge items and 4 engagement items. The remaining items were modified as described above.

#### Integrating input from science- and practice-based experts

From the content validity assessment, we added 11 knowledge items and 7 engagement items that we hypothesized fit within existing domains. New knowledge items related to stakeholders’ perceived understanding of the following concepts: systems approaches and innovative solutions (included in the *intervention factors* domain); nontraditional partnerships (*roles*); aligning community priorities, translating ideas into action, facilitators of implementation, and barriers of implementation (*sustainability*, which we subsequently renamed *implementation & sustainability*); social costs of obesity, inequities, and social determinants of health (*problem*); and building on community assets and strengths (*resources*). New items related to stakeholders’ engagement with childhood obesity prevention efforts included: inclusivity (*dialogue* *&* *mutual learning*), adapting to changing conditions (*flexibility*), shared decision-making power and building relationships with key influencers (*influence & power*), shared community ownership and long-term commitment (*leadership & stewardship*), and trustworthiness (*trust*, which we subsequently renamed *trust & trustworthiness*). Eleven of the 18 new items reflect concepts salient to both science- and practice-based experts, whereas the remaining items were salient to one group (6 items from science-based experts and 1 item from practice-based experts). As described below, confirmatory factor analysis tested the structural fit of these items and domains.

### Field test and assessment of measurement properties

#### Sample characteristics

The refined release candidate (v3) survey with 25 knowledge items and 28 engagement items was fielded among stakeholders in three U.S. communities: Greenville County, South Carolina (*n*=120 invitations, *n*=51 responses, *n*=50 complete knowledge/engagement responses); East Boston, Massachusetts (*n*=61 invitations, *n*=34 responses, *n*=30 complete knowledge/engagement responses); and Tucson, Arizona (total invitations sent unknown, *n*=93 responses, *n*=84 complete knowledge/engagement responses). The total analytic sample included *n*=164 complete responses. Fourteen of the 19 core group members in Greenville County (73.7%) completed the retest survey a median 13 days after completion of the initial survey (range 10–17 days).

Table 2 reports participants’ demographic characteristics overall and by community. Most participants were female (72.6% overall, range 56.7–82.0% across communities) did not have a Hispanic or Latino background (80.5% overall, range 72.6–96.0%), reported White race only (79.3% overall, range 73.3–82.0%), and had a Master’s degree or higher level of education (62.2% overall, range 56.7–72.0%). Participants were on average 46.4 (SD 11.5) years old.

**Table 2:** Demographic characteristics among field test participants, overall and by community, 2019–2020

	Overall	Greenville County, SC	East Boston, MA	Tucson, AZ
Analytic sample size, *n*^a^	164	50	30	84
**Characteristic**				
Age (years), mean (SD)	46.4 (11.5)	49.5 (10.7)	40.9 (10.3)	46.3 (11.8)
No response, *n* (%)	10 (6.1)	0 (0)	2 (6.7)	8 (9.5)
Gender, *n* (%)				
Female	119 (72.6)	41 (82.0)	17 (56.7)	61 (72.6)
Male	34 (20.7)	9 (18.0)	11 (36.7)	14 (16.7)
No response	11 (6.7)	0 (0)	2 (6.7)	9 (10.7)
Hispanic or Latino, *n* (%)				
Yes	22 (13.4)	2 (4.0)	5 (16.7)	15 (17.9)
No	132 (80.5)	48 (96.0)	23 (76.7)	61 (72.6)
No response	10 (6.1)	0 (0)	2 (6.7)	8 (9.5)
Race, *n* (%)				
White only	130 (79.3)	41 (82.0)	22 (73.3)	67 (79.8)
Black or African American only	11 (6.7)	8 (16.0)	2 (6.7)	1 (1.2)
Other^b^	11 (6.7)	1 (2.0)	2 (6.7)	8 (9.5)
No response	12 (7.3)	0 (0)	4 (13.3)	8 (9.5)
Education level, *n* (%)				
Less than college degree	10 (6.1)	3 (6.0)	3 (10.0)	4 (4.8)
College degree	41 (25.0)	11 (22.0)	7 (23.3)	23 (27.4)
Master’s degree or higher	102 (62.2)	36 (72.0)	17 (56.7)	49 (58.3)
No response	11 (6.7)	0 (0)	3 (10.0)	8 (9.5)

^a^The analytic sample included those with complete knowledge and engagement responses. As reported above, some respondents did not report complete demographic information

^b^The Other race category included respondents selecting American Indian or Alaska Native, Asian, Native Hawaiian or Pacific Islander, multiple races, and other

We used CFA to test hypothesized 5-factor measurement models of both knowledge and engagement scales. Model fit indices of the initial and modified models are reported in Table 3 for both knowledge and engagement scales. We also report the following for the modified scales: factor correlations (Table 4), descriptive statistics and reliability characteristics (Table 5), and item descriptive statistics and item-factor loadings (Additional File 2).

**Table 3:** Confirmatory factor analysis model fit for proposed and modified knowledge and engagement scales (*n*=164)

	Knowledge		Engagement	
	Proposed 5-factor model	Modified 4-factor model^b^	Proposed 5-factor model	Modified 5-factor model
**# items**	25	23	28	23
**Fit criteria**^a^				
*χ*^2^ (df) (↓ better)	438.3 (265)	346.7 (224)	508.0 (340)	317.1 (220)
RMSEA (90% CI) (upper CI < 0.80)	0.063 (0.052, 0.074)	0.058 (0.046, 0.069)	0.055 (0.045, 0.065)	0.052 (0.039, 0.064)
SRMR (< 0.08)	0.075	0.068	0.063	0.055
CFI (≥ 0.95)	0.848	0.878	0.865	0.914
TLI (≥ 0.95)	0.828	0.863	0.850	0.902

^a^Hu L, Bentler PM. Cutoff criteria for fit indexes in covariance structure analysis: Conventional criteria versus new alternatives. Structural equation modeling: a multidisciplinary journal. 1999;6 (1):1-55.

*χ*^*2*^ *(df)* chi-square test (degrees of freedom); *RMSEA* root mean square error of approximation (< 0.05 = good fit and upper CI < 0.08); *SRMR* standardized root mean square residual (< 0.05 = good fit and < 0.08 = adequate fit); *CFI* Comparative Fit Index (> 0.95 = good fit and > 0.90 = adequate fit); *TLI* Tucker-Lewis Index (> 0.95 = good fit and > 0.90 = adequate fit)

^b^*Roles* merged with *resources* factor in modified knowledge scale

**Table 4:** Correlations among factors in modified 23-item knowledge and 23-item engagement scales (*n*=164)

Knowledge	1	2	3	4	Engagement	1	2	3	4	5
1. *Intervention factors*	-	0.82	0.82	0.53	1. *Dialogue & mutual learning*	-	0.86	0.74	0.87	0.72
2. *Roles & resources*		-	0.87	0.55	2. *Flexibility*		-	0.63	0.79	0.66
3. *Implementation & sustainability*			-	0.60	3. *Influence & power*			-	0.69	0.58
4. *Problem*				-	4. *Leadership & stewardship*				-	0.77
					5. *Trust & trustworthiness*					-

Correlations are from standardized results of the confirmatory factor analysis

**Table 5:** Subscale descriptive statistics and reliability characteristics for modified 23-item knowledge and 23-item engagement scales

	# items	Mean (SD)	α	ICC	WSCV (95% CI)
Analytic sample size, *n*	-	164	164	14	14
**Knowledge**					
1. *Intervention factors*	5	3.6 (0.8)	0.87	0.50	0.14 (0.10, 0.21)
2. *Roles & resources*	7	3.5 (0.7)	0.87	0.86	0.08 (0.05, 0.11)
3. *Implementation & sustainability*	6	3.5 (0.7)	0.86	0.78	0.09 (0.07, 0.14)
4. *Problem*	5	4.2 (0.6)	0.87	0.62	0.09 (0.06, 0.13)
**Engagement**					
1. *Dialogue & mutual learning*	4	3.7 (0.9)	0.88	0.87	0.09 (0.06, 0.13)
2. *Flexibility*	4	3.9 (0.5)	0.75	0.87	0.07 (0.05, 0.10)
3. *Influence & power*	4	3.2 (0.9)	0.87	0.95	0.07 (0.05, 0.11)
4. *Leadership & stewardship*	8	3.8 (0.7)	0.90	0.96	0.03 (0.02, 0.05)
5. *Trust & trustworthiness*	3	4.0 (0.6)	0.80	0.70	0.08 (0.06, 0.12)

*CI* confidence interval, *ICC* intraclass correlation coefficient, *SD* standard deviation, *WSCV* within-subject coefficient of variation. ICC estimates were based on multiple observers and two-way mixed-effects models and are commonly interpreted as < 0.40 “poor”; 0.40–0.59 “fair”; 0.60–0.74 “good”; 0.75–1.00 “excellent”. WSCV estimates are interpreted as the average percentage of variation between test and retest scores

#### Knowledge scale

In the proposed 5-factor model, the *roles* factor had correlations approaching 1 with *intervention factors* (0.97), *implementation & sustainability* (0.94), and *resources* (0.93)—an indication that these factors may be combined. Conceptually, stakeholders’ roles in childhood obesity prevention efforts can be viewed as a human resource (consistent with the Resources’ dimension of the Community Readiness Model [24]), and therefore, we tested model fit with *roles* and *resources* factors merged together (hereafter labeled *roles & resources*). With this change, overall model fit improved (Table 3) and standardized item factor loadings remained high for *roles & resources* (ranging between 0.65 and 0.78). Additionally, we eliminated one item from the *intervention factors* domain (“I am knowledgeable about risk factors related to childhood obesity”) due to high mean baseline response with lower response variability (4.5±0.6 points) and lower factor loading (Additional File 2). We also eliminated one item from the *implementation & sustainability* domain (“I am knowledgeable about strategies (like practices, programs, policies) to prevent childhood obesity that will have the greatest impact in promoting healthy weight in our children”) due to high correlation with the subsequent item about sustaining strategies over time (Spearman correlation=0.82) and conceptually poorer fit relating to effectiveness rather than implementation processes.

With these changes, the modified knowledge scale included 23 items across four factors. Correlations among factors ranged from 0.53 (*intervention factors* and *problem*) to 0.87 (*roles & resources* and *implementation & sustainability*) (Table 4). The four subscales had strong internal scale consistency (each *α*=0.9) (Table 5). Stakeholders had the lowest mean knowledge scores for *roles & resources* and *implementation & sustainability* subscales (each 3.5±0.7 points) and highest scores for the *problem* subscale (4.2±0.6 points) (Table 5). Subscale scores were not significantly different across communities (Additional File 3). ICC estimates suggested “fair” test-retest absolute agreement for the *intervention factors* subscale scores, “good” agreement for the *problem* subscale scores, and “excellent” agreement for the *roles & resources* and *implementation & sustainability* subscale scores [23]. WSCVs ranged from 0.08 (*roles & resources*) to 0.14 (*intervention factors*) (Table 5).

#### Engagement scale

In reviewing CFA output from the proposed 5-factor model and item characteristics, we eliminated five engagement items. From the *dialogue & mutual learning* domain, we eliminated two items (“I pay attention to what colleagues say about childhood obesity prevention in [community]” and “I can openly discuss problems related to childhood obesity prevention in [community]”), each due to high baseline response with lower response variability (4.1±0.7 and 4.0±0.8 points, respectively) and the latter having a lower factor loading (Additional File 2). We eliminated two items from the *leadership & stewardship* domain (“I am motivated to prevent childhood obesity in [community]” and “I have good skills for working with other people and organizations that are preventing childhood obesity in [community]”)—the first due to high baseline response with lower variability (4.4±0.7 points) and high correlation with an item about “long-term commitment” (Spearman correlation=0.72), and the second due to high baseline response with lower variability (4.1±0.7 points) and lower factor loading (Additional File 2). Lastly, we eliminated one item from the *trust & trustworthiness* domain (“I trust others involved in childhood obesity prevention efforts in [community]”) due to its lower factor loading and low correlation with the total engagement scale (0.35).

With these changes, the modified engagement scale included 23 items across five factors. Factor correlations ranged from 0.58 (*influence & power* and *trust & trustworthiness*) to 0.87 (*dialogue & mutual learning* and *leadership & stewardship*) (Table 4). The five subscales had strong internal scale consistency with Cronbach’s *α* values between 0.8 and 0.9 (Table 5). Mean subscale scores were lowest for *influence & power* (3.2±0.9 points) and highest for *trust & trustworthiness* (4.0±0.6 points) (Table 5). *Trust & trustworthiness* subscale scores were significantly different across communities (*p*=0.03), in which the Tucson mean score (3.8±0.6 points) was lower than that of Greenville County and East Boston (each 4.1±0.6 points). (Additional File 3). ICC estimates suggested “good” test-retest absolute agreement for *trust & trustworthiness* scores and “excellent” agreement for other subscale scores [23]. WSCVs ranged from 0.03 (*leadership & stewardship*) to 0.09 (*dialogue* *& mutual learning*) (Table 5).

## Discussion

The COMPACT Stakeholder-driven Community Diffusion Survey measures three stakeholder characteristics that we hypothesize are important for the planning, implementation, and sustainability of whole-of-community interventions aiming to prevent childhood obesity: stakeholders’ perceived *knowledge* of childhood obesity and how to address it, their *engagement* with the issue, and their *social networks*. Building on our prior survey development work (which was limited to reliability testing only) [5], we assessed validity and further refined the knowledge and engagement measures. This multi-method process yielded modified 23-item knowledge and 23-item engagement scales that reflect concepts salient to science- and practice-based experts. Results from the psychometric evaluation increase our confidence in the modified scales’ construct validity based a four-factor structure for knowledge (*roles & resources* merged) and a five-factor structure for engagement. Findings also suggest strong internal scale consistency with Cronbach’s *α* for knowledge and engagement subscales at or above 0.75. Additionally, ICC estimates indicate “good” or “excellent” 2-week test-retest agreement for most subscale scores, with within-subject variation ranging between 8% and 14% for knowledge subscales and between 3% and 9% for engagement subscales.

In our prior work, knowledge and engagement concepts were conceived, vetted, and pilot-tested by an international team of investigators with deep expertise in whole-of-community childhood obesity prevention interventions [5]. However, through the content validity assessments in the current study, we took an important next step in incorporating a broader range of expertise from both researcher and practitioner perspectives. Participants in both expert groups gave strong ratings of the existing knowledge and engagement domains, contributing to our confidence that the scales reflect important stakeholder characteristics that might help catalyze community change. Yet beyond this confirmatory finding, the assessments yielded key additions to our knowledge and engagement measures that span concepts like systems approaches, social determinants of health, inclusivity, shared decision-making power, and shared community ownership. With these changes, we believe that the revised survey instrument will have greater resonance with its users and reflect critical, up-to-date focal points of what might be required to address childhood obesity at the community-level.

Although some variability is expected across diverse community settings and contexts, we observed minor to no statistical differences in mean knowledge and engagement subscale scores between stakeholders in three U.S. communities at each project’s respective baseline assessment (Additional File 3). Differences in knowledge and engagement trajectories over time, particularly those that coincide with distinct implementation efforts like leadership development and resource sharing, would increase our confidence of the measures’ predictive validity.

Response variability and ceiling effects are challenges of many measures that use a Likert scale [25]. As such, one of our primary survey refinement goals was to increase knowledge and engagement item response variability, while also keeping an eye toward decreasing participant burden. When refining the survey, we conservatively eliminated three beta prototype (v2) items specifically due to limited response variability during prior projects (Additional File 1). In response to cognitive response testing, we also modified item language to promote a greater range of answers—for example, by anchoring all engagement items to childhood obesity prevention concepts rather than broad individual characteristics like being flexible and a strong leader. Analysis of the refined release candidate (v3) survey items demonstrated stronger response variability with many item SDs ≥ 1.0 points and all ≥ 0.6 points of the 5-point scale. In the final modification process moving toward the stable release (v4) survey, we eliminated 7 additional items (2 knowledge and 5 engagement) that had lower response variability among other criteria (e.g., lower factor loading). The resulting knowledge and engagement scores are lower than those previously reported [5]—perhaps an indication of the measures’ potential to assess meaningful change over time.

The modified knowledge and engagement scales demonstrated strong reliability characteristics, with multiple indicators exceeding those reported from our prior testing with the beta prototype (v2) survey [5]. Internal scale consistency improved for all knowledge subscales (e.g., for *intervention factors*, from *α*=0.58 previously to *α*=0.87 in our current study). Engagement internal scale consistency was high in both studies; however, we observed stronger 2-week test-retest agreement for each engagement subscale score (e.g., for *influence & power*, from ICC=0.55 and 13% within-subject variation to ICC=0.95 and 7% within-subject variation).

In their current form, the knowledge and engagement measures are intended to be used by applied researchers working with communities to address childhood obesity. However, a key next step is to adapt this research tool for community practice. We envision a web-based platform that streamlines survey administration, analysis, and interpretation—essentially creating an evidence-based decision-making tool for community stakeholders that offers targeted resources, recommendations, and potential courses of action. Community stakeholders could use the tool to help recognize (and subsequently monitor) potential areas of growth, for example, in their understanding of available resources to address childhood obesity, perceived influence in affecting policy or practice change, and in alignment with the Stakeholder-driven Community Diffusion theory of change—awareness of how these knowledge and engagement characteristics are distributed in their networks [5–9]. Stakeholders may subsequently allocate time and resources toward these “knowledge and engagement targets,” such as professional development opportunities including training in Community-based System Dynamics [20, 26], continuing education units, advocacy training, and other capacity building activities throughout their networks.

### Study strengths and limitations

Study strengths include our comprehensive, multi-method study design to achieve numerous survey validation and refinement goals. A couple of limitations should be considered. First, new survey items added as a result of the content validity assessments were not tested for cognitive response due to timing with the field tests. Testing revised items for their cognitive response is a priority of future research, and for the knowledge *intervention factors* subscale especially, improving item comprehension may help strengthen the observed “fair” test-retest reliability. At this juncture, we have incorporated feedback from community partners to simplify survey language and improve readability (Additional File 2). Second, while most psychometric properties were evaluated among the full sample of stakeholders in three U.S. communities, due to feasibility constraints, we assessed test-retest reliability among a small sample of stakeholders in one community only. However, baseline scores were similar across communities (Additional File 3). Due to the convenience sampling approach for multiple study phases (cognitive response testing, test-retest reliability), results may not be generalizable to other community stakeholder populations.

## Conclusions

Findings from this multi-method survey development process increase our confidence of the COMPACT Stakeholder-driven Community Diffusion knowledge and engagement measures’ content validity, reliability, and construct validity. Future research is required to assess the measures’ predictive validity of intervention implementation and sustainability.

## Supplementary information

**Additional file 1: **Annotated knowledge and engagement survey items: beta prototype (v2) to release candidate (v3) changes and rationale

**Additional file 2:.** Knowledge and engagement survey item characteristics from the field test (n=164 stakeholders in three communities), 2019-2020

**Additional file 3:.** Subscale scores stratified by community for modified 23-item knowledge and 23-item engagement scales

**Additional file 4: **STROBE Statement—Checklist of items that should be included in reports of ***cross-sectional studies***

## Data Availability

Data are not available due to participants’ consent to keep data with the research team only.

## Abbreviations

CI: Confidence interval
CFA: Confirmatory factor analysis
CFI: Comparative Fit Index
COMPACT: Childhood Obesity Modeling for Prevention and Community Transformation
ICC: Intraclass correlation coefficient
RMSEA: Root mean square error of approximation
SD: Standard deviation
SRMR: Standardized root mean square residual
TLI: Tucker-Lewis Index
WSCV: Within-subject coefficient of variation
