# Supplementary material for: Validation and refinement of the Stakeholder-driven Community Diffusion Survey for childhood obesity prevention
Source: Implement Sci. 2021 Oct 9;16:91. doi: 10.1186/s13012-021-01158-4 (PMC8501696; doi:10.1186/s13012-021-01158-4)
Supplement: Supplementary file 1 — Additional file 1: Annotated knowledge and engagement survey items: beta prototype (v2) to release candidate (v3) changes and rationale [file 13012_2021_1158_MOESM1_ESM.docx]

# ADDITIONAL FILE 1

# Annotated knowledge and engagement survey items: *beta prototype* (v2) to *release candidate* (v3) changes and rationale

**Notes:**

1. *Beta prototype* (v2) response options: strongly disagree, disagree, neutral, agree, strongly agree.
2. *Release candidate* (v3) response options: strongly disagree, disagree, neither agree nor disagree, agree, strongly agree.
3. All *release candidate* (v3) items are anchored to childhood obesity prevention concepts, as we expect that they will be more sensitive to change over time (rather than general statements about leadership abilities, for example) and more specific/actionable intervention targets for coalition work.
4. All *release candidate* (v3) knowledge items have the same stem (*I am knowledgeable about…*) and all engagement items begin with *I*…
5. We reviewed *beta prototype* (v2) items using the following criteria derived from extant measurement research:^1-5^
   - *Limited response variability* in > 1 measurement period in both community sites, defined conservatively as a SD < 10% of the maximum response value.
   - *Limited change* in mean scores (< 5%) across measurement rounds.
   - *Low item-total correlations* (< 0.4) in both community sites.

Items are highlighted in yellow if they met the any of the above criteria and were considered for elimination from the survey. However, if an item captured concepts salient in the content validity assessment, then it was retained. Items that were eliminated are ~~highlighted in yellow and crossed out~~ (4 knowledge items and 4 engagement items).

1. New *release candidate* (v3) items based on concepts from the content validity assessments are shown in **bold blue** (11 knowledge items and 7 engagement items).

________________________

^1^Boateng GO, Neilands TB, Frongillo EA, Melgar-Quiñonez HR, Young SL. Best Practices for Developing and Validating Scales for Health, Social, and Behavioral Research: A Primer. Front Public Health. 2018;6:149.

^2^Sapsford R. Constructing scales. Survey Research. 2nd ed. London: SAGE Publications, Ltd; 2007. p. 222-45.

^3^Revicki D, Hays RD, Cella D, Sloan J. Recommended methods for determining responsiveness and minimally important differences for patient-reported outcomes. Journal of Clinical Epidemiology. 2008;61(2):102-9.

^4^Hays RD, Hadorn D. Responsiveness to change: an aspect of validity, not a separate dimension. Quality of Life Research. 1992;1(1):73-5.

^5^Fok CCT, Henry D. Increasing the Sensitivity of Measures to Change. Prevention Science: The Official Journal of the Society for Prevention Research. 2015;16(7):978-86.

**KNOWLEDGE (18 🡪 25 items)**

**Knowledge Domain 1: *Intervention factors* (6 🡪 6 items)**

|  | ***Beta prototype* (v2)** | **Notes** | ***Release candidate* (v3)**  *I am knowledgeable about…* | **Notes** |
| --- | --- | --- | --- | --- |
| ~~k1_1~~ | Preventing obesity early in life is important | Limited response variability and change over time; low item-total correlation | [item eliminated] |  |
| ~~k1_2~~ | Certain populations of children are more vulnerable to obesity | Limited response variability and change over time; low item-total correlation | [item eliminated] | Concept supported by both expert groups; see new item (k4_5) in the *Problem* domain |
| k1_3 | I feel confident in listing risk factors related to early childhood obesity |  | …risk factors related to childhood obesity |  |
| k1_4 | I am aware of evidence-based strategies that target risk factors related to early childhood obesity |  | …evidence-based strategies that target risk factors related to childhood obesity |  |
| k1_5 | I feel confident in listing possible policy changes to combat early childhood obesity in [community] |  | …possible policy changes to prevent childhood obesity in [community] |  |
| k1_6 | I feel confident in suggesting changes in early childhood settings (like child care, medical settings, or play areas) to prevent early childhood obesity |  | …possible changes in childhood settings (like schools, medical settings, play areas) to prevent childhood obesity in [community] |  |
| **k1_7** | - |  | …how to use systems approaches to prevent childhood obesity in [community] | Concept supported by both expert groups |
| **k1_8** | - |  | …how to innovate new strategies to prevent childhood obesity in [community] | Concept supported by science-based experts |

*Beta prototype* (v2) response options: strongly disagree, disagree, neutral, agree, strongly agree.

*Release candidate* (v3) response options: strongly disagree, disagree, neither agree nor disagree, agree, strongly agree.

**Knowledge Domain 2: *Roles* (3 🡪 3 items)**

|  | ***Beta prototype* (v2)** | **Notes** | ***Release candidate* (v3)**  *I am knowledgeable about…* | **Notes** |
| --- | --- | --- | --- | --- |
| k2_1 | I can play a role in preventing early childhood obesity in [community] |  | …my role in preventing childhood obesity in [community] |  |
| k2_2 | I know what is being done in [community] to prevent early childhood obesity |  | …what is being done by others in [community] to prevent childhood obesity |  |
| ~~k2_3~~ | Obesity prevention strategies should be prioritized and implemented by multiple early childhood providers | Limited change over time | [item eliminated] |  |
| **k2_4** | - |  | …how to create nontraditional partnerships (for example, with food businesses or the private sector) to prevent childhood obesity in [community] | Concept supported by both expert groups |

*Beta prototype* (v2) response options: strongly disagree, disagree, neutral, agree, strongly agree.

*Release candidate* (v3) response options: strongly disagree, disagree, neither agree nor disagree, agree, strongly agree.

**Knowledge Domain 3: *Sustainability* (3 🡪 7 items) *renamed as *implementation & sustainability****

|  | ***Beta prototype* (v2)**  *I know strategies to prevent obesity in childhood that…* | **Notes** | ***Release candidate* (v3)**  *I am knowledgeable about strategies (like practices, programs, policies) to prevent childhood obesity that…* | **Notes** |  |
| --- | --- | --- | --- | --- | --- |
| k3_1 | …will be acceptable and appropriate for [community] | Double-barreled item | …will be appropriate for [community] | “Appropriate” supported by both expert groups (e.g., strategies appropriate for different cultures and population groups) |  |
| k3_2 | …will have the greatest impact in promoting healthy weight |  | …that will have the greatest impact in promoting healthy weight |  |  |
| k3_3 | …can be sustained over time |  | …that can be sustained over time (for many years in the future) |  |  |
|  | | | *I am knowledgeable about…* |  | |
| **k3_4** | - |  | …how to align childhood obesity prevention efforts with other community priorities in [community] | Concept supported by both expert groups |  |
| **k3_5** | - |  | …how to translate ideas about preventing childhood obesity into action | Concept supported by practice-based experts |  |
| **k3_6** | - |  | …the barriers to implementing childhood obesity prevention strategies in [community] | Concept supported by science-based experts |  |
| **k3_7** | - |  | …the facilitators of implementing childhood obesity prevention strategies in [community] | Concept supported by science-based experts |  |

*Beta prototype* (v2) response options: strongly disagree, disagree, neutral, agree, strongly agree.

*Release candidate* (v3) response options: strongly disagree, disagree, neither agree nor disagree, agree, strongly agree.

**Knowledge Domain 4: *Problem* (3 🡪 5 items)**

|  | ***Beta prototype* (v2)** | **Notes** | ***Release candidate* (v3)**  *I am knowledgeable about…* | **Notes** |
| --- | --- | --- | --- | --- |
| ~~k4_1~~ | Early childhood obesity is a problem in Somerville | Limited change over time | [item eliminated] |  |
| k4_2 | There are increased health care costs due to medical complications of obesity in early childhood | Limited change over time; unclear if item refers to short-term or long-term healthcare costs | …the economic costs (like healthcare spending, disability, absenteeism) related to obesity throughout the life course | Concept supported by both expert groups |
| k4_3 | I am familiar with other illnesses and health concerns associated with obesity in early childhood | Unclear if item refers to health issues in early childhood or later in life | …the health concerns (like cardiovascular disease, some cancers, depression) associated with obesity throughout the life course |  |
| **k4_4** | - |  | …the social costs (like weight stigma, decreased quality of life) related to obesity throughout the life course | Concept supported by both expert groups |
| **k4_5** | - |  | …the existing racial, ethnic, and/or socioeconomic health inequities related to childhood obesity in [community] | Concept supported by both expert groups |
| **k4_6** | - |  | …the social determinants of health (like education, healthcare, housing) related to childhood obesity in [community] | Concept supported by both expert groups |

*Beta prototype* (v2) response options: strongly disagree, disagree, neutral, agree, strongly agree.

*Release candidate* (v3) response options: strongly disagree, disagree, neither agree nor disagree, agree, strongly agree.

**Knowledge Domain 5: *Resources* (3 🡪 4 items)**

|  | ***Beta prototype* (v2)** | **Notes** | ***Release candidate* (v3)**  *I am knowledgeable about…* | **Notes** |
| --- | --- | --- | --- | --- |
| k5_1 | I know where to find resources related to early childhood obesity prevention |  | …where to find resources related to childhood obesity prevention in [community] |  |
| k5_2 | I am aware of staff education or training opportunities that include the subject of early childhood obesity prevention |  | …professional development, staff education, or training opportunities related to childhood obesity prevention in [community] | Included “professional development” per input from science-based experts |
| k5_3 | I am aware of evaluation and monitoring efforts in [community] that address childhood obesity in young children |  | …evaluation and monitoring efforts in [community] that address childhood obesity |  |
| **k5_4** | - |  | …how to build on [community]’s assets and strengths to prevent childhood obesity | Concept supported by both expert groups |

*Beta prototype* (v2) response options: strongly disagree, disagree, neutral, agree, strongly agree.

*Release candidate* (v3) response options: strongly disagree, disagree, neither agree nor disagree, agree, strongly agree.

**ENGAGEMENT (25 🡪 28 items)**

**Engagement Domain 1: *Dialogue & mutual learning* (7 🡪 6 items)**

|  | ***Beta prototype* (v2)** | **Notes** | ***Release candidate* (v3)** | **Notes** |
| --- | --- | --- | --- | --- |
| ~~e1_1~~ | I can talk openly and honestly at work or meetings | Limited change over time; concept supported by both expert groups, but item dropped due to similarity with e1_6 | [item eliminated] |  |
| e1_2 | I make an effort to participate in discussions |  | I make an effort to participate in discussions about childhood obesity prevention in [community] |  |
| ~~e1_3~~ | I listen to colleagues when someone expresses a concern | Limited response variability; item conceptually similar to e1_4 (and highly correlated) | [item eliminated] |  |
| e1_4 | I am attentive to what colleagues say when they speak |  | I am attentive to what colleagues say when they speak about childhood obesity prevention in [community] |  |
| e1_5 | I share my ideas and suggestions whether or not colleagues agree with my input |  | I share my ideas and suggestions about childhood obesity prevention whether or not colleagues agree with my input |  |
| e1_6 | I can openly discuss problems and issues |  | I can openly discuss problems and issues related to childhood obesity prevention in [community] |  |
| e1_7 | I work with colleagues to develop the best possible approach to our work |  | I work with colleagues to develop the best possible approach to our work related to childhood obesity prevention in [community] |  |
| **e1_8** | - |  | I facilitate a sense of inclusivity that engages diverse individuals and groups working to prevent childhood obesity in [community]^a^ | Concept supported by both expert groups |

*Beta prototype* (v2) response options: strongly disagree, disagree, neutral, agree, strongly agree.

*Release candidate* (v3) response options: strongly disagree, disagree, neither agree nor disagree, agree, strongly agree.

^a^ Adapted from the Internal Coalition Effectiveness instrument: Cramer ME, Atwood JR, Stoner JA. Measuring community coalition effectiveness using the ICE instrument. *Public Health Nurs.* 2006;23(1):74-87.

**Engagement Domain 2: *Flexibility* (3 🡪 4 items)**

|  | ***Beta prototype* (v2)** | **Notes** | ***Release candidate* (v3)** | **Notes** |
| --- | --- | --- | --- | --- |
| e2_1 | I am willing to make compromises related to my work in childhood obesity prevention |  | I am willing to make compromises related to my work in childhood obesity prevention |  |
| e2_2 | I work to come up with solutions that satisfy all colleagues |  | I work to come up with solutions related to childhood obesity prevention that satisfy all colleagues |  |
| e2_3 | I respect different points of view from colleagues | Limited change over time; concept salient in both expert groups | I encourage mutual respect for different perspectives related to childhood obesity prevention |  |
| **e2_4** | - |  | I am able to adapt to changing conditions (like fewer funds than expected, change in political climate or in leadership) to prevent childhood obesity in [community]^a^ | Concept supported by science-based experts |

*Beta prototype* (v2) response options: strongly disagree, disagree, neutral, agree, strongly agree.

*Release candidate* (v3) response options: strongly disagree, disagree, neither agree nor disagree, agree, strongly agree.

^a^ Adapted from Mattessich P, Murray-Close, M., Monsey, B. Wilder Collaboration Factors Inventory. In. St. Paul, MN: Amherst H. Wilder Foundation; 2001. This item was initially included in the *alpha prototype* (v1) survey and was rated highly by COMPACT investigators when the 50-item engagement scale was assessed for content validity in 2015. The item was eliminated prior to the *beta prototype* (v2) due to low test-retest reliability in the retrospective pilot testing.

**Engagement Domain 3: *Influence & power* (2 🡪 4 items)**

|  | ***Beta prototype* (v2)** | **Notes** | ***Release candidate* (v3)** | **Notes** |
| --- | --- | --- | --- | --- |
| e3_1 | I influence decisions that affect childhood obesity prevention efforts in [community] |  | I influence decisions that affect childhood obesity prevention efforts in [community] |  |
| e3_2 | I influence the policies and actions of community-based childhood obesity prevention |  | I influence policies and actions related to childhood obesity prevention in [community] |  |
| **e3_3** | - |  | I promote shared decision-making power related to childhood obesity prevention efforts in [community]^a^ | Concept supported by science-based experts |
| **e3_4** | - |  | I build strategic relationships with key influencers (like elected officials, funders) that can impact childhood obesity prevention efforts in [community] | Concept supported by science-based experts |

*Beta prototype* (v2) response options: strongly disagree, disagree, neutral, agree, strongly agree.

*Release candidate* (v3) response options: strongly disagree, disagree, neither agree nor disagree, agree, strongly agree.

^a^ Item supported by: (i) Schulz AJ, Israel BA, Lantz P. Instrument for evaluating dimensions of group dynamics within community-based participatory research partnerships. *Evaluation and Program Planning.* 2003;26(3):249-262 and (ii) Calancie L, Allen NE, Weiner BJ, Ng SW, Ward DS, Ammerman A. Food Policy Council Self-Assessment Tool: Development, Testing, and Results. *Prev Chronic Dis.* 2017;14:E20.

**Engagement Domain 4: *Leadership & stewardship* (10 🡪 10 items)**

|  | ***Beta prototype* (v2)** | **Notes** | ***Release candidate* (v3)** | **Notes** |
| --- | --- | --- | --- | --- |
| e4_1 | I am motivated to prevent childhood obesity |  | I am motivated to prevent childhood obesity in [community] |  |
| e4_2 | My passion and enthusiasm for childhood obesity prevention motivates others |  | I motivate others with my passion and enthusiasm to prevent childhood obesity in [community] |  |
| e4_3 | I establish positive relationships with community members with whom my colleagues want to engage and mobilize |  | I establish positive relationships with community members with whom my colleagues want to engage and mobilize to prevent childhood obesity in [community] |  |
| e4_4 | I have good skills for working with other people and organizations | Limited change over time; concept salient in both expert groups | I have good skills for working with other people and organizations that are preventing childhood obesity in [community] |  |
| ~~e4_5~~ | I lead by example | Limited change over time; misinterpreted by interview respondents as relating to personal health behaviors | [item eliminated] |  |
| ~~e4_6~~ | I encourage colleagues to express their opinions and thoughts | Limited change over time; concept salient in both expert groups, but item dropped due to similarity with *Dialogue & mutual learning* domain and items | [item eliminated] |  |
| e4_7 | I emphasize the importance of having a collective sense of mission | Limited change over time; concept salient in both expert groups | I emphasize the importance of having a collective sense of mission to prevent childhood obesity in [community] |  |
| e4_8 | I provide leadership and guidance in maintaining relationships among colleagues |  | I provide leadership and guidance in maintaining relationships among colleagues working to prevent childhood obesity in [community] |  |
| e4_9 | I advocate strongly for my own opinions and agendas |  | I advocate strongly for my own opinions and agendas related to childhood obesity prevention efforts in [community] |  |
| e4_10 | I do not give up when faced with challenges |  | I do not give up when faced with challenges related to childhood obesity prevention in [community] |  |
| **e4_11** | - |  | I encourage community ownership of efforts to prevent childhood obesity in [community] | Concept supported by both expert groups |
| **e4_12** | - |  | I have long-term commitment to preventing childhood obesity in [community] | Concept supported by both expert groups |

*Beta prototype* (v2) response options: strongly disagree, disagree, neutral, agree, strongly agree.

*Release candidate* (v3) response options: strongly disagree, disagree, neither agree nor disagree, agree, strongly agree.

**Engagement Domain 5: *Trust* (3 🡪 4 items) *renamed as *trust* *& trustworthiness****

|  | ***Beta prototype* (v2)** | **Notes** | ***Release candidate* (v3)** | **Notes** |
| --- | --- | --- | --- | --- |
| e5_1 | I trust others involved in childhood obesity prevention efforts | Limited change over time; concept supported by both expert groups | I trust others involved in childhood obesity prevention efforts in [community] |  |
| e5_2 | People involved in childhood obesity prevention efforts trust me |  | I think that people involved in childhood obesity prevention efforts in [community] trust me | Changed to “I statement” for consistency |
| e5_3 | I try to promote a climate of collaboration and trust | Limited change over time; concept supported by both expert groups | I am effective in promoting a climate of trust among colleagues working to prevent childhood obesity in [community]^a^ |  |
| **e5_4** | - |  | I can be counted on when working to prevent childhood obesity in [community]^b^ | Item added to capture another aspect of trustworthiness; concept supported by both expert groups |

*Beta prototype* (v2) response options: strongly disagree, disagree, neutral, agree, strongly agree.

*Release candidate* (v3) response options: strongly disagree, disagree, neither agree nor disagree, agree, strongly agree.

^a^ Wording change in alignment with original item from: National Cancer Institute Science of Team Science (SciTS) Team. Interpersonal Collaboration Scale. https://www.teamsciencetoolkit.cancer.gov/public/TSResourceMeasure.aspx?tid=2&rid=391. Accessed February 14, 2020.

^b^ Adapted from the CDC Partnership Trust Tool Survey: https://orphroadmap.org/docs/modernization/Partnership_Trust_Tool.pdf

**Correspondence:**

Ariella R. Korn, PhD, MPH

Cancer Prevention Fellow, Implementation Science Team

Division of Cancer Control and Population Sciences

National Cancer Institute, National Institute of Health

9609 Medical Center Drive, Rockville, MD 20850

Email: ariella.korn@nih.gov
