# Supplementary material for: Validation and refinement of the Stakeholder-driven Community Diffusion Survey for childhood obesity prevention
Source: Implement Sci. 2021 Oct 9;16:91. doi: 10.1186/s13012-021-01158-4 (PMC8501696; doi:10.1186/s13012-021-01158-4)
Supplement: Supplementary file 2 — Additional file 2:. Knowledge and engagement survey item characteristics from the field test (n=164 stakeholders in three communities), 2019-2020 [file 13012_2021_1158_MOESM2_ESM.docx]

ADDITIONAL FILE 2

Knowledge and engagement survey item characteristics from the field test (n=164 stakeholders in three communities), 2019-2020

Note: track changes on survey items indicate modifications used in Tucson, Arizona in response to community partners’ feedback to improve readability. These changes are recommended for survey applications moving forward.

Knowledge

| **Knowledge domain** | **Knowledge item**  *I am knowledgeable about…* | **Mean (SD)** | **Median (range)** | **Standardized factor loading of modified 23-item scale**^a^ |
| --- | --- | --- | --- | --- |
| **1. *Intervention factors*** | …risk factors related to childhood obesity^b^ | 4.5 (0.6) | 5 (2, 5) | - |
|  | …evidence-based strategies that target risk factors related to childhood obesity | 4.0 (0.9) | 4 (1, 5) | 0.67 |
|  | …possible policy changes to prevent childhood obesity in [community] | 3.4 (1.0) | 4 (1, 5) | 0.71 |
|  | …possible changes in childhood settings (like schools, medical settings, play areas) to prevent childhood obesity in [community] | 3.8 (0.9) | 4 (1, 5) | 0.74 |
|  | …how to use systems approaches to prevent childhood obesity in [community] | 3.5 (1.0) | 4 (1, 5) | 0.86 |
|  | …how to innovate new strategies to prevent childhood obesity in [community] | 3.4 (1.0) | 4 (1, 5) | 0.84 |
| **2. *Roles & resources***  *domains merged in modified 23-item scale | …my role in preventing childhood obesity in [community] | 4.0 (0.9) | 4 (2, 5) | 0.70 |
|  | …what is being done by others in [community] to prevent childhood obesity | 3.4 (0.9) | 4 (1, 5) | 0.65 |
|  | …how to create partnerships outside of my sector (for example, with food businesses or the private sector) to prevent childhood obesity in [community] | 3.5 (1.0) | 4 (1, 5) | 0.67 |
|  | …where to find resources (like programs, tools, materials) related to childhood obesity prevention in [community] | 3.6 (1.0) | 4 (2, 5) | 0.67 |
|  | …professional development, education, or training opportunities related to childhood obesity prevention in [community] | 3.3 (1.0) | 3 (2, 5) | 0.74 |
|  | …evaluation and monitoring efforts in [community] that address childhood obesity | 3.1 (1.0) | 3 (1, 5) | 0.78 |
|  | …how to build on [community]’s strengths to prevent childhood obesity | 3.4 (0.9) | 3 (1, 5) | 0.74 |
| **3. *Implementation & sustainability*** | …strategies (like practices, programs, policies) to prevent childhood obesity that will be appropriate for [community] | 3.6 (0.9) | 4 (1, 5) | 0.76 |
|  | …strategies (like practices, programs, policies) to prevent childhood obesity that will have the greatest impact in promoting healthy weight in our children^c^ | 3.5 (1.0) | 4 (1, 5) | - |
|  | …strategies (like practices, programs, policies) to prevent childhood obesity that can last over time (for many years in the future) | 3.4 (1.0) | 4 (1, 5) | 0.77 |
|  | …how to line up childhood obesity prevention efforts with other priorities in [community] | 3.3 (0.9) | 3.5 (1, 5) | 0.67 |
|  | …how to turn ideas about preventing childhood obesity into action | 3.7 (0.8) | 4 (2, 5) | 0.67 |
|  | …factors that make it harder to carry out childhood obesity prevention strategies in [community] (“barriers”) | 3.8 (0.9) | 4 (1, 5) | 0.68 |
|  | …factors that make it easier to carry out childhood obesity prevention strategies in [community] (“facilitators”) | 3.4 (0.9) | 4 (1, 5) | 0.72 |
| **4. *Problem*** | …economic costs (like healthcare spending, disability, work absences) related to obesity throughout the life course | 3.9 (1.0) | 4 (1, 5) | 0.73 |
|  | …health concerns (like cardiovascular disease, some cancers, depression) related to obesity throughout the life course | 4.4 (0.7) | 4 (2, 5) | 0.75 |
|  | …social costs (like weight stigma, decreased quality of life) related to obesity throughout the life course | 4.2 (0.7) | 4 (2, 5) | 0.71 |
|  | …racial, ethnic, and/or socioeconomic health inequities related to childhood obesity in [community] | 4.2 (0.7) | 4 (2, 5) | 0.79 |
|  | …social determinants of health (like education, healthcare, housing) related to childhood obesity in [community] | 4.2 (0.8) | 4 (2, 5) | 0.86 |

^a^ All standardized factor loadings are statistically significant with *p* < 0.001.

^b^ Item eliminated due to high baseline value, limited response variability, and lower factor loading.

^c^ Item eliminated due to high correlation with subsequent item (Spearman correlation = 0.82) and conceptually poorer fit relating to “impact” rather than implementation processes.

Engagement

| **Engagement domain** | **Engagement item** | **Mean (SD)** | **Median (range)** | **Standardized factor loading of modified 23-item scale**^a^ |
| --- | --- | --- | --- | --- |
|  |  |  |  |  |
| **1. *Dialogue & mutual learning*** | I try to participate in discussions about childhood obesity prevention in [community] | 3.6 (1.0) | 4 (1, 5) | 0.80 |
|  | I pay attention to what colleagues say about childhood obesity prevention in [community]^b^ | 4.1 (0.7) | 4 (2, 5) | - |
|  | I share my ideas about childhood obesity prevention whether or not colleagues agree with me | 3.8 (1.0) | 4 (1, 5) | 0.74 |
|  | I can openly discuss problems related to childhood obesity prevention in [community]^c^ | 4.0 (0.8) | 4 (2, 5) | - |
|  | I work with colleagues to develop the best approach to our work related to childhood obesity prevention in [community] | 3.6 (1.1) | 4 (1, 5) | 0.86 |
|  | I promote a sense of belonging to engage diverse individuals and groups working to prevent childhood obesity in [community] | 3.8 (0.9) | 4 (1, 5) | 0.81 |
| **2. *Flexibility*** | I am willing to make compromises related to my work in childhood obesity prevention | 3.9 (0.7) | 4 (2,5) | 0.56 |
|  | I work to come up with solutions related to childhood obesity prevention that satisfy all colleagues | 3.5 (0.9) | 4 (1, 5) | 0.74 |
|  | I encourage mutual respect for different perspectives related to childhood obesity prevention | 4.2 (0.7) | 4 (3, 5) | 0.64 |
|  | I can adapt to changing conditions (like fewer funds than expected, change in political climate or leadership) to prevent childhood obesity in [community] | 4.1 (0.7) | 4 (2, 5) | 0.65 |
| **3. *Influence & power*** | I influence decisions that affect childhood obesity prevention efforts in [community] | 3.1 (1.1) | 3 (1, 5) | 0.91 |
|  | I influence policies related to childhood obesity prevention in [community] | 2.9 (1.1) | 3 (1, 5) | 0.88 |
|  | I promote shared decision-making related to childhood obesity prevention efforts in [community] | 3.6 (1.0) | 4 (1, 5) | 0.70 |
|  | I build strategic relationships with influential people (like elected officials, funders) that can impact childhood obesity prevention efforts in [community] | 3.4 (1.1) | 4 (1, 5) | 0.65 |
| **4. *Leadership & stewardship*** | I am motivated to prevent childhood obesity in [community]^d^ | 4.4 (0.7) | 5 (2, 5) | - |
|  | I motivate others with my enthusiasm to prevent childhood obesity in [community] | 3.7 (1.0) | 4 (1, 5) | 0.85 |
|  | I form positive relationships with community members with whom my colleagues want to engage and mobilize to prevent childhood obesity in [community] | 4.0 (0.8) | 4 (1, 5) | 0.76 |
|  | I have good skills for working with other people and organizations that are preventing childhood obesity in [community]^e^ | 4.1 (0.7) | 4 (1, 5) | - |
|  | I emphasize the importance of having a collective sense of mission to prevent childhood obesity in [community] | 4.0 (0.9) | 4 (1, 5) | 0.79 |
|  | I provide leadership and guidance in maintaining relationships among colleagues working to prevent childhood obesity in [community] | 3.5 (1.0) | 4 (1, 5) | 0.76 |
|  | I advocate for my own opinions and agendas related to childhood obesity prevention efforts in [community] | 3.2 (0.9) | 3 (1, 5) | 0.60 |
|  | I do not give up when faced with challenges related to childhood obesity prevention in [community] | 3.7 (0.8) | 4 (2, 5) | 0.67 |
|  | I encourage community ownership of efforts to prevent childhood obesity in [community] | 3.8 (0.8) | 4 (1, 5) | 0.65 |
|  | I am committed to preventing childhood obesity in [community] in the long-term | 4.2 (0.9) | 4 (1, 5) | 0.73 |
| **5. *Trust & trustworthiness*** | I trust others involved in childhood obesity prevention efforts in [community]^f^ | 4.1 (0.6) | 4 (3, 5) | - |
|  | I think that people involved in childhood obesity prevention efforts in [community] trust me | 3.9 (0.7) | 4 (2, 5) | 0.67 |
|  | I promote trust among colleagues working to prevent childhood obesity in [community] | 3.9 (0.7) | 4 (2, 5) | 0.80 |
|  | I can be counted on when working to prevent childhood obesity in [community] | 4.1 (0.7) | 4 (2, 5) | 0.81 |

^a^ All standardized factor loadings are statistically significant with *p* < 0.001.

^b^ Item eliminated due to high baseline value and limited response variability.

^c^ Item eliminated due to high baseline value, limited response variability, and lower factor loading.

^d^ Item eliminated due to high baseline value, limited response variability, and high correlation with “long-term commitment” item (Spearman correlation = 0.72).

^e^ Item eliminated due to high baseline value, limited response variability, and lower factor loading.

^f^ Item eliminated due to lower factor loading and low correlation with total scale (0.35).

**Correspondence:**

Ariella R. Korn, PhD, MPH

Cancer Prevention Fellow, Implementation Science Team

Division of Cancer Control and Population Sciences

National Cancer Institute, National Institute of Health

9609 Medical Center Drive, Rockville, MD 20850

Email: ariella.korn@nih.gov
