# Supplementary material for: Validation and refinement of the Stakeholder-driven Community Diffusion Survey for childhood obesity prevention
Source: Implement Sci. 2021 Oct 9;16:91. doi: 10.1186/s13012-021-01158-4 (PMC8501696; doi:10.1186/s13012-021-01158-4)
Supplement: Supplementary file 3 — Additional file 3:. Subscale scores stratified by community for modified 23-item knowledge and 23-item engagement scales [file 13012_2021_1158_MOESM3_ESM.docx]

ADDITIONAL FILE 3

Subscale scores stratified by community for modified 23-item knowledge and 23-item engagement scales

|  | **# items** | **Greenville County, SC** | **East Boston, MA** | **Tucson, AZ** |
| --- | --- | --- | --- | --- |
| Analytic sample size, n |  | 50 | 30 | 84 |
| **Knowledge** |  |  |  |  |
| 1. *Intervention factors* | 5 | 3.7 (0.9) | 3.5 (0.8) | 3.6 (0.8) |
| 2. *Roles & resources*^a^ | 7 | 3.6 (0.8) | 3.5 (0.6) | 3.4 (0.7) |
| 3. *Implementation & sustainability* | 6 | 3.5 (0.9) | 3.5 (0.6) | 3.5 (0.6) |
| 4. *Problem* | 5 | 4.1 (0.7) | 4.2 (0.7) | 4.2 (0.6) |
| **Engagement** |  |  |  |  |
| 1. *Dialogue & mutual learning* | 4 | 3.7 (0.9) | 3.6 (0.9) | 3.7 (0.8) |
| 2. *Flexibility* | 4 | 3.8 (0.6) | 4.0 (0.5) | 3.9 (0.5) |
| 3. *Influence & power* | 4 | 3.4 (0.9) | 3.3 (1.0) | 3.1 (0.8) |
| 4. *Leadership & stewardship* | 8 | 3.7 (0.7) | 3.8 (0.7) | 3.8 (0.6) |
| 5. *Trust & trustworthiness*^b^ | 3 | 4.1 (0.6) | 4.1 (0.6) | 3.8 (0.6) |

^a^ *Roles* domain merged with *resources* domain in modified scale.

^b^ *Trust & trustworthiness* domain scores were significantly different across communities (*p* = 0.03) using the Kruskal-Wallis rank sum test. Other scores were not significantly different across communities.

**Correspondence:**

Ariella R. Korn, PhD, MPH

Cancer Prevention Fellow, Implementation Science Team

Division of Cancer Control and Population Sciences

National Cancer Institute, National Institute of Health

9609 Medical Center Drive, Rockville, MD 20850

Email: ariella.korn@nih.gov
